# Pain Medicine in Crisis—A Possible Model toward a Solution: Empowering Community Medicine to Treat Chronic Pain

**DOI:** 10.5041/RMMJ.10134

**Published:** 2013-10-29

**Authors:** Amir Minerbi, Simon Vulfsons

**Affiliations:** 1 Department of Family Medicine, Clalit Health Services, Haifa and Western Galilee District, Israel;; 2 Institute for Pain Medicine, Rambam Health Care Campus, Haifa, Israel

**Keywords:** Chronic pain, crisis in pain medicine, postgraduate medical training, primary care, secondary care

## Abstract

Pain medicine in Israel and in the world has reached a crisis. The lack of available pain medicine services is resulting in the unsatisfactory treatment for chronic pain sufferers. The main causes of this crisis are: 1) the high prevalence of chronic pain, reaching levels of 17% in the adult population;2) the lack of appropriate training of primary care physicians in the field of chronic pain management; and 3) the paucity of consultation services in the field of chronic pain.

In this journal article, we propose a possible model for the solution of the problem, based upon levels of treatment according to the severity of the disease and upon training of primary and secondary care physicians in the treatment of pain. According to the model, the vast majority of treatment and management will take place in the community after appropriate training of primary care physicians. More complex cases will be referred to secondary care community-based pain clinics manned by physicians with further in-depth training. Only the most complex of patients, or those needing specialized treatment such as invasive analgesic therapy, will be referred to tertiary pain centers manned by specialists in pain medicine.

Implementation of this model will necessitate training of primary care physicians and the establishment of secondary care facilities and can, in our opinion, pose a pragmatic solution for the hundreds of thousands of patients suffering from chronic pain.

## INTRODUCTION

Pain is often defined as “an unpleasant sensory and emotional experience.” 1 This experience is common to everyone as almost all of us experience pain throughout our lives. Since the birth of humanity, medical science has strived to alleviate pain.

Chronic pain is significantly different to acute pain not only in its physiological characteristics but also in the emotional and social consequences that are associated with it, such as mood disturbances, decreased quality of life, loss of productivity, and increased utilization of medical resources. 2 – 8 The high prevalence of chronic pain adds an additional burden to the bio-psycho-social aspect of the phenomenon. According to a study by Breivik published in 2006, 17% of the adult Israeli population suffers from chronic pain. 9 Other authors have reported an even higher prevalence of pain. 10

With such a high prevalence rate and its serious consequences, it should come as no surprise that pain in general, and especially chronic pain, leads to high visitation rates with physicians. It is well documented that pain is second only to respiratory symptoms as the primary reason for patients to visit their doctor. 11 Patients suffering from chronic pain are often referred to various specialists and some to pain clinics. These clinics, in Israel and throughout the world, may have waiting lists of many months. The long waiting causes further suffering and a continuing deterioration in the patients’ quality of life. 12

The huge demand for the services of pain clinics lies far above their ability to supply this demand. This problem is definitely not singular to Israel. Researchers from around the world have reported on similar disproportionate supply for the demand of specialist pain medicine services. The average time on the waiting list in Australia is 5 months, 13 in Spain 6 months, and in Canada 3 months to 5 years. 14 There is no argument that these waiting list times are far too long, but how long should the waiting list time for a pain specialist consultation be? There are very few data to suggest an answer to this question; however, in England, for example, a waiting list time of 13 weeks has been defined to be maximal. 15 In Canada a task force has been set up to decide upon the maximal acceptable waiting list time. 14

Until a solution is found, the problem of patients suffering from chronic pain will remain well within the realm of the family practitioner, frustrated by the lack of appropriate resources to treat these patients and ease their suffering. 16 A solution to this problem that has been deemed by the World Health Organization as a health crisis of primary priority 6 calls for a new approach. In this article, we will describe the crisis in which the pain clinics in Israel (and worldwide) have found themselves from epidemiological, medical, and economic viewpoints. We will offer a possible solution based upon multi-tiered intervention and the empowerment of community medical teams treating chronic pain patients. We will also present data of the initial 3 years of implementing this model in the Rambam School of Pain Medicine.

## THE CAUSES FOR THE CRISIS IN PAIN MEDICINE IN ISRAEL

The world crisis facing pain medicine stems from the high prevalence of chronic pain, but its severity is augmented by the insufficient treatment of chronic pain in the community in combination with the low availability of pain consultation.

The paucity of pain consultation services is striking: in Israel only 50 physicians are board-certified in pain medicine, and approximately 20 others work predominantly in this field. The discrepancy between the small number of pain physicians and the huge number of pain patients is striking. The burden of treating pain patients therefore lies almost entirely on the shoulders of community primary care physicians. But the solution given by community-based medicine is often unsatisfactory. In a survey conducted among family practitioners, barriers to effective treatment of pain were reported, including lack of consultation services (77% of the responses), lack of knowledge (64% of the responses), and concern about the use and side effects of opiate drugs. 16 The long waiting lists for pain clinic services result in many doctors ceasing to refer to these clinics. 5 , 17 Thus we can see that many physicians in the primary care setting feel that they lack adequate clinical skills in treating chronic pain. Why?

In a review published in 2008 about the failure of the treatment of patients suffering from chronic pain in Britain, the authors state that the main reason for this lies with inadequate education in pain in the medical schools’ curriculum. 18 Similar findings have been reported in the United States, 19 Canada, 20 and in Britain. 21

During their years as medical students future family practitioners receive almost no tools for dealing with pain. During their years of residency the gaps are not narrowed. Pain as an independent topic is not part of the formal education of the family residency program in Israel, 22 although topics are studied in various rotations, such as orthopedics, neurology, and rheumatology. Even so, the training of family practitioners in Israel does not give them adequate tools for managing patient suffering from chronic pain.

Thus the pain medicine crisis stems from the very high prevalence of chronic pain coupled with poor training in the primary care setting and no secondary center consultant services. It is obvious that the vast scope of this phenomenon does not afford a solution that can be based upon tertiary pain centers. The key to the solution lies in the hands of community-based medicine. 16

The crisis is reminiscent of that faced by the primary care community a few years ago with the outbreak of the “diabetes epidemic.” At that time, the dramatic increase in patients suffering from diabetes mellitus brought about an overflow in the number of patients in the diabetes clinics and a deterioration in their treatment. 23 The similarity between the case for diabetes and the case for chronic pain is striking: Both conditions are chronic, the prevalence high and increasing with age, and they cause severe morbidity and a decrease in quality of life. In both diseases treatment can rely on equipment and medications readily found in the community setting. The realization that the challenge of the diabetic epidemic could not be adequately met in the tertiary care centers brought about the implementation of a project aimed at moving the treatment out of the hospitals and into the family practitioners’ clinics. In order to achieve this, the family practitioners underwent training that empowered them with the necessary knowledge and tools; thus they became the leaders in the treatment of diabetes. The consultant diabetes clinics were then able to allocate more time to complicated patients, while coordinating with the family practitioners as effective partners. 24

We would like to propose a model for the solution of the pain crisis, based upon the stratification of patient allocation according to the severity of their condition. This model will involve primary, secondary, and tertiary clinics empowered with the necessary knowledge and skills for managing the patients in the appropriate tier of care.

## THE RATIONALE FOR A SOLUTION TO THE CRISIS—BUILDING A NEW PAIN MANAGEMENT SYSTEM

The crisis in the management of patients with chronic pain stems from three major problems: the high prevalence of chronic pain, the lack of knowledge in the management of chronic pain by primary care physicians, and the poor availability of pain consultancy services.

The high prevalence rates suggest that any attempt to solve the pain crisis based upon increasing the availability of tertiary care services is doomed, by weight of numbers, to fail. Thus the only viable solution to the crisis is empowerment of primary care physicians, especially family practitioners, who can be taught effective skills in chronic pain management.

**Figure 1:**
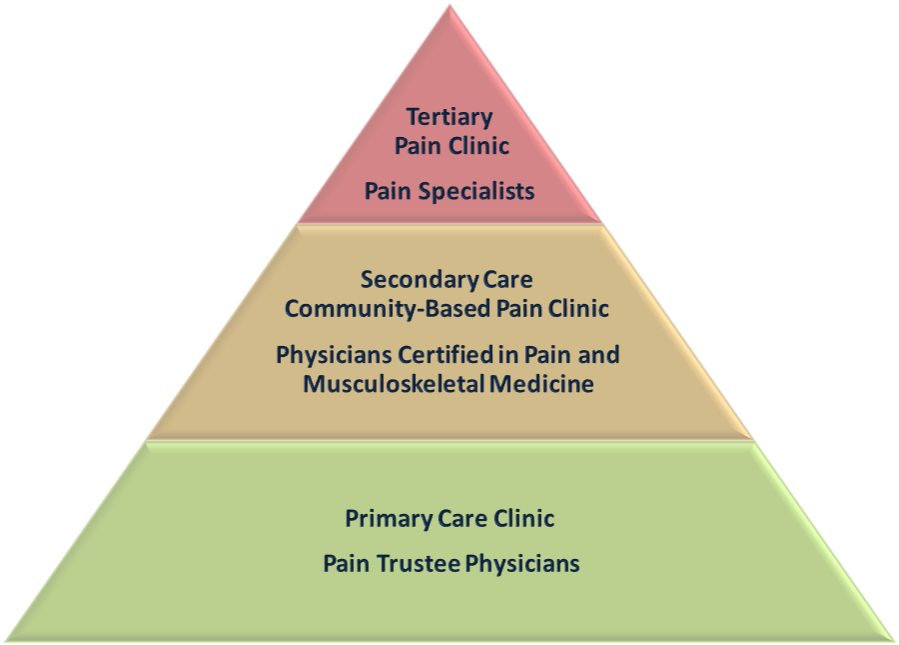
**
The Pyramid Model for the Stratification of Chronic Pain Treatment in the Community.
** Most pain patients will be treated by primary care physicians trained as pain trustees. More challenging patients will be treated by physicians certified in pain and musculoskeletal medicine in secondary community clinics, while the most severe patients and the ones requiring invasive procedures will be seen in tertiary pain clinics. The two bottom levels of the pyramid—training medical students and addressing the public—are not discussed in this paper and have been omitted from the figure.

The model presented below is based upon empowering primary care physicians with education stressing theoretical knowledge together with practical hands-on clinically oriented learning. The model constitutes a three-tiered pyramid, each tier narrower than the one below, from the lower-tiered primary care physicians who should be trained as pain trustees, up to the second-tiered secondary care or community consultant physicians, and up to the tertiary center-based specialists in pain medicine ( Figure 1 ). In our model, the vast majority of pain patients should be treated in the primary care setting. Patients suffering from more complex problems should be referred to secondary, community-based consultants, probably working within a small multidisciplinary setup. These consultants could either be pain specialist working in the community or primary care physicians who have undergone further training and are pain trustees with an a additional diploma in pain and musculoskeletal medicine. Finally, the top tier should be populated by specialists in pain medicine who have undergone extensive further training, especially in the utilization of invasive procedures. Only the most complex of cases, and those needing specialized care, should be referred to tertiary pain centers.

The main challenge in realizing this model lies in the training of doctors according to these tiers, empowering them with knowledge and skills necessary for the task. There are already a few year-long courses being taught in Israel, most notably in the Rambam School of Pain Medicine in association with the Technion, at Tel Aviv University in association with the family practice association, and at the Beer Sheva School of Medicine. In the following section we will give a detailed description of the necessary training of the various levels (see Figure 1 and Table 1 ).

**Table 1:** Operative Plan for Implementing the Pyramid Model.

** Pyramid Level **	** Definition **	** The Vision **	** Operative Steps to Realize the Vision **

Primary care physicians in the community—“pain trustees”	Generally family practitioners but also orthopedic surgeons and neurologists	Will know how to manage the pain of the majority of patients in the community by a “mechanism-derived pain medicine” and “bio-psycho-social” approach	Advanced training during residency or in a school of pain medicine Upon completion of pain training will receive a certificate as “pain trustee”
Secondary care physicians—pain and musculoskeletal medicine diploma-certified	Physicians who have undergone advanced training and who consult for the primary care physicians in their catchment area	Will coordinate pain problems in their area Will have advanced training and skills	Studies toward a diploma of 300 hours’ theoretical and practical training Upon completion of studies will receive a certificate, “Diploma in Pain and Musculoskeletal Medicine”
Tertiary care pain specialists	Physicians who are board-certified in pain medicine	Will complete a full board certified pain medicine residency program Will be able to coordinate pain management and teaching at all levels	Training in a recognized certified pain program

## PRIMARY CARE PHYSICIANS

Patients who suffer chronic pain are primarily seen by family physicians and, to a lesser extent, by orthopedic surgeons, neurologists, rheumatologists, and other specialists. Since most of these physicians have not received sufficient training in the treatment of pain as students and as residents, their knowledge in the field is based primarily on postgraduate education. And indeed the knowledge in pain medicine is unsatisfactory not only in Israel but also worldwide. 19 , 25 The training of community orthopedists poses a unique problem, since their training is surgery-oriented, while most of the musculoskeletal problems encountered in the community call for conservative, bio-psycho-socially oriented management. 26 Until recent years, pain training programs were few and offered mainly theoretical teaching, with little practical training. In recent years more pain training courses have become available, and several pain medicine schools are currently active, training primary care physicians.

We are aware that many primary care physicians will have less interest in pain and musculoskeletal problems than in diabetes mellitus and metabolic syndrome for example, but we are convinced that a basic working knowledge of the diagnosis and treatment of patients suffering from pain necessitates further postgraduate training even for those with less interest. We propose that most of the primary care physicians undergo theoretical and practical training, thus becoming qualified pain trustees.

### Vision for Community Primary Care Physicians

Community primary care physicians will receive continuous theoretical and practical training in pain medicine both during their residency and as continued medical education. Primary physicians will be able to take pain mechanism-based history, perform a focused physical and neurological examination, and manage the treatment of patients’ pain based on the bio-psycho-social model, including referral to imaging studies, tailoring appropriate pharmacotherapy, and orchestrating other pain treatment modalities: physical therapy, mental health services, and pain clinics.

## SECONDARY CARE PHYSICIANS, CERTIFIED IN COMMUNITY PAIN MEDICINE

All fields of medicine benefit from community-based medical experts. Pain medicine as well would benefit from certified physicians serving as secondary referral addresses, mid-way between primary care medicine and tertiary pain centers. To date, few such secondary pain clinics are available in Israel, which are naturally unable to address the needs of the myriad patients in pain. The residency program in pain medicine, which opened recently, is not expected to solve the problem adequately due to the enormous gap between number of residents (around 10 nationally) and the national need of pain specialists (estimated in hundreds). We therefore suggest that the solution lies in the training of primary care physicians in pain medicine, which should take place in two steps: first, basic training as pain trustees, and then diploma studies in pain medicine. At the end of the 300-hour training program, including theoretical and practical training, certified physicians will be awarded a diploma in pain and musculoskeletal medicine. The extent of the training program as well as its requirements is in accord with the specifications published by the International Federation of Musculoskeletal Medicine. 27

Having completed their training program, secondary care physicians will be able to run multi-disciplinary regional community-based pain clinics, treat patients referred from primary clinics, and refer some patients to the tertiary clinics. Secondary care physicians will enjoy the professional support of the tertiary pain centers. They will subsequently be able to tutor other primary care physicians undertaking pain medicine training.

### Vision for Pain-certified Secondary Care Physicians

Certified secondary care physicians are the professional backbone of pain treatment in the community. They are certified in pain and musculoskeletal medicine, having gained extensive knowledge and proficiency in the field. They are able to manage the care of a large part of community patients in pain and master several therapeutic techniques. They are also trained to work in a multidisciplinary approach and to collaborate with other care givers such as manual therapists, psychologists, and others.

## TERTIARY PAIN CENTER PHYSICIANS

These physicians, specialists and residents in pain medicine, are the professional head of the pyramid. They practice their specialty, diagnosing, treating, researching, and teaching pain medicine. As part of their work they collaborate with secondary care and tertiary pain clinics.

### Vision for Tertiary Pain Center Physicians

These physicians are of the highest level of skill and training in treating patients in pain, in medical knowledge and experience, in research, and finally in teaching pain medicine to care givers in all levels of the pyramid. They aim to improve the treatment of pain through improved diagnosis and treatment, medical research, and teaching.

In addition to the three levels of the pyramid described in the previous paragraphs, we consider highly important the optimization of pain medicine training given to all physicians. We suggest this be done by implementing a structured educational program in pain medicine, which will be a part of the formal syllabus of all faculties of medicine.

## THE RAMBAM SCHOOL OF PAIN MEDICINE MODEL (SEE APPENDIX)

Since October 2010, three yearlong programs have been completed involving 80 physicians and 2 dentists ( Table 2 ). A fourth program started in October 2013 with 27 primary care physicians and 12 nurses. Each program has consisted of 18 bi-weekly, 6 academic hour meetings. The major incentive of these programs for the physicians has been their desire to gain knowledge and skills in dealing with patients suffering from pain. Up till now they have felt inadequate in treating pain patients in their family practice. Course content includes 50% hands-on training with clinical laboratories. A few of the participants come from health medical organizations that reimburse a modest fee (approximately 40NIS or 12USD) for trigger point therapy. Participants who completed the courses received a certificate of “Pain Trustee,” testifying to 108 hours of education in pain medicine. In October 2012 we held, in addition, a second-year course for 16 practitioners who had satisfactorily completed the first year. This course ran for 144 hours in total (24 meetings of 6 hours), together with a practicum of an additional 48 hours. The two courses, totaling 300 hours, entitled the participants to the certificate of “Diploma in Pain and Musculoskeletal Medicine.” The second-year course was 75% practical hands-on. The certificates were given in conjunction with the Technion (Israel Institute of Technology) School for Continuing Medical Education.

**Table 2:** Participants in Pain Trustee and Diploma Courses 2010–2013.

** Year **	** Course **	** Participants **	** Certified **	** Comments **

2010–2011	Pain trustees	29 (including 1 dentist)	22	108 hours (18X6)
2011–2012	Pain trustees	23 (including 1 dentist)	20	108 hours (18X6)
2012–2013	Pain trustees	25	23	108 hours (18X6)
2012–2013	Diploma in pain and musculoskeletal medicine	16	16	144 hours (24X6)
2013–2014	Pain trustees	38 (including 12 nurses)		120 hours (20X6) Started October 2013

## PRACTICAL IMPLICATIONS

### Planning

In Israel, the first steps, in the form of courses in pain medicine, were initiated by knowledgeable physicians with a keen interest in empowering the primary care family practitioners. This spontaneous “bottom-up” implementation is, in our minds, insufficient in order to propel a nationwide project. We suggest that a “top-down” approach is necessary as well. This implies planning and implementation directed by the senior medical and financial administration. By this approach, the needs of the medical system realize strategic planning, taking into account such aspects as outcome definition, planning stages, fund allocation, and quality control. All these are lacking in the Israeli medical system concerning chronic pain. We recommend utilizing the energy and the need that manifests from ground level and the experience and knowledge from physicians dedicated to teaching and training in pain medicine in order to build a program based upon vision, planning, and implementation. Realizing such a program necessitates allocation of resource (see below) that would be supplied by the Ministry of Health and the health care funds ( *sherutei briut* ). Thus in our view it is logical to incorporate top-down decision-making with bottom-up activity in a unified model.

### Budget

The extensive educational programs for primary care physicians that have taken place in the last few years in Israel have been heavily subsidized by pharmaceutical and medical equipment companies. In addition, there has been some very modest sponsoring by a few of the health care funds for some of the physicians attending these programs (personal information, S.V.).

If we assume educating 100 primary care physicians in Israel in yearlong “pain trustee” courses, together with an additional 20 physicians who will participate in a second year culminating in a Diploma in Pain and Musculoskeletal Medicine, the funding needed will be approximately 800,000 shekels a year for the whole country (personal information, S.V. Director of the Rambam School of Pain Medicine). Fund allocation for the actual treatment of patients suffering from pain in the community setting will be minimal, as patients suffering from chronic pain are treated, in the most part, by primary care physicians. 9 The modest costs of setting up secondary care pain clinics will most probably be offset by avoiding the unnecessary cost of the poor and wasteful treatment experienced today by most patients treated by the undertrained physicians today. For the medical system that spends millions of shekels on poor treatment today, these costs are modest.

### Evaluation and Quality Control

Realization of the pyramid model that conceptualizes transfer of the onus of management of chronic pain patients from the tertiary centers back to the community necessitates a deep change in the training of physicians at the various levels of treatment. The efficacy of this move must be evaluated with measurable parameters. One obvious parameter could be the change in waiting list time for pain clinics, although this might not necessarily reflect on the quality of care given in the community. We recommend the following parameters that would give evidence of a change in the bio-psycho-social aspects of the chronic pain phenomena: 1) in the biological aspect we could follow such parameters as VAS and the use of pain medication 28 , 29 ; 2) on the psychological aspect one could examine quality of life measures such as patient satisfaction, stress, anxiety, and anger 30 , 31 ; and 3) such social parameters as days off work and physician visitation rates.

### Economic and Financial Considerations

More effective treatment for chronic pain should have economic advantages such as decreased requests for imaging, decreased referral to consultant services, and decreased pharmaceutical spending. 31 These also are measurable parameters.

Finally, since this model is expected to be implemented gradually, it would be possible to compare areas that have implemented the model to areas that have not yet reached implementation stages.

### Incentive of Physicians to Participate in the Training Program

The program relies on the voluntary participation of primary care physicians and furthermore on their willingness to pay for the training. One may wonder what incentive these physicians would have to undertake such an effort. We suggest a few such incentives: Firstly, primary physicians are often frustrated by their inability to help pain patients (and these constitute a significant part of daily visits); by acquiring relevant skills they may enhance their ability to help patients and their sense of self competence. Secondly, many primary care physicians seek professional horizons that would enable them to devote part of their job to specific fields of medicine. Thirdly, the skills acquired in the program will not only attract new patients to join the physicians’ clinic, but would also be applicable in the private practice.

And indeed, for these reasons among others, we see an impressive demand for the training programs offered in Israel, the extent of which outstrips the supply of pain schools.

### Other Concerns

The training of primary care physicians would necessitate a paradigm shift in the way patients suffering from pain are dealt with in the community. Pain trustees and especially secondary pain medicine diplomates will need to coordinate with other services, especially in the field of physical medicine such as physiotherapy, osteopathy, chiropractors, etc., as well as psychosocial services. The secondary care physicians are suitably trained and positioned to facilitate the proposed multidimensional service and will need additional administrative staff to maintain it.

This has already taken place in six centers in Israel with physicians that have graduated from our courses running a multidisciplinary, community-based service.

## CONCLUSION

Pain relief medicine both in Israel and worldwide is experiencing a deep crisis that results in inadequate availability of pain relief services to the enormous number of patients suffering from chronic pain. The extent of the crisis is reflected by the long waiting lists for pain relief services. Among the reasons for the crisis are the high prevalence of chronic pain leading to a huge demand for pain relief services, the lack of simple definitive treatments, the paucity of pain specialists, and the insufficient knowledge in the treatment of chronic pain among primary care physicians.

The above-suggested solution is based on the empowerment of primary care physicians, by providing them with tools that would enable them to treat most chronic pain patients in the community. The pyramid model suggests a tiered approach to the patient in pain, graded by the gravity of their condition. Most patients will be treated by primary care pain trustee physicians, more complex patients will be treated by pain and musculoskeletal certified physicians in secondary care clinics, and only the most complicated patients and those who require invasive procedures will be treated by pain specialists in tertiary care centers.

This model, whose realization is already taking its first steps, will necessitate conceptual and financial support. We believe its implementation may reduce the load on pain clinics, reduce the frustration of primary care physicians faced with chronic pain patients, and—most importantly—will relieve the distress of hundreds and thousands of patients in Israel whose suffering is currently unanswered.

## Supplementary Materials


