# Supplementary material for: Pain Medicine in Crisis—A Possible Model toward a Solution: Empowering Community Medicine to Treat Chronic Pain
Source: Rambam Maimonides Med J. 2013 Oct 29;4(4):e0027. doi: 10.5041/RMMJ.10134 (PMC3820300; doi:10.5041/RMMJ.10134)
Supplement: Supplementary file 1 [file rmmj-4-4-e0027-s001.pdf]

This appendix has been provided by the authors to provide readers with additional background reading and information about their work

Appendix: The Rambam School of Pain Medicine

Appendix to Minerbi A, Vulfsons S. Pain Medicine in Crisis—A Possible Model toward a Solution: Empowering Community Medicine to Treat Chronic Pain. Rambam Maimonides Med J 2013;4 (4):e0027. doi:10.5041/RMMJ.10134

The Rambam School of Pain Medicine was established in January 2010 under the direction of the second author, Dr Simon Vulfsons, who at that time was the deputy director of the Rambam Institute for Pain Medicine. The vision of the school was to improve health care for patients suffering from chronic pain by supplying up-to-date relevant teaching to physicians and allied professionals dealing with these patients. The main focus was and is to improve the knowledge and skill of primary care physicians, the main group bearing the brunt of treating the vast numbers of patients suffering from chronic pain. The scope of teaching was defined as postgraduate level. Undergraduate teaching, especially of medical students, is not part of the scope of this school.

The mission was to design and implement courses that not only lay down theoretical foundations of care but also supply hands-on skills that can be applied in the physician's primary care clinic, with minimal extra cost and no extra infrastructure.

THE PAIN TRUSTEE COURSE

The course was designed to provide, over a year, the large bulk of learning in pain medicine with the following basic content:

a. **Basic training:** This covers all the core elements of assessing a patient in pain, including detailed history-taking and relevant physical examination with emphasis on assessing both pain and functional status.

b. **Clinical laboratories:** For these, the participants bring in their own patients who then undergo a complete pain intake with one of the instructors and a group of up to eight course participants. These laboratories, lasting 90 minutes, have proven to be essential for the participants to hone their skills, and we have managed to diagnose and treat many patients in these sessions.

c. **Learning hands-on skills:** We have spent approximately 20% of the course teaching needle and injection techniques.

d. **Problem-based learning sessions:** Most frontal learning is performed with a problem-based approach, allowing the participants to interact with each other and with the tutor.

e. **Case reporting (40% of the final grade):** Each participant is requested to supply five short case reports and one long report as part of the course requirements. The cases are read, commented on, and sent back to the participant for feedback.

f. **Examinations (60% of the final grade):** There are three examinations throughout the course, each having equal weight of 20%.

Participants who complete the course with attendance of 85% or above and 65 on their final accumulative grade are awarded a certificate as “pain trustees.” While this certificate has no extrinsic value at present, we are convinced that in the future all primary care physicians will have to complete courses equivalent to the basic pain trustee level.

THE DIPLOMA IN PAIN AND MUSCULOSKELETAL MEDICINE COURSE

This is a second-year course designed to increase the knowledge and skills of those physicians who wish to develop their pain practice and eventually become secondary tier pain medicine providers. The course is much longer (144 hours) and has a 75% hand-on component, with an additional 25% in frontal lectures. This course was run during 2012–2013, and all 16 participants received their diplomas. In-depth mini-courses were incorporated into the course material, such as strain–counter-strain techniques, sports medicine clinics with stabilizing and taping techniques, and advanced needle techniques. There was also an extensive real-time clinic run by the participants under the guidance of the tutors.

Practicum

All graduates of the courses (both first and second year) can apply for a minimum 50-hour practicum at the Rambam Institute of Pain Medicine. In the practicum sessions, participants can be directly involved in a real-time running clinic where patients are being treated. There are various levels of practicum, from observation only to semi-autonomous treatment by participants under supervision. We are now expanding the number of sites where a practicum can be performed.

Teaching year 2013–2014

This year, due to great demand, we decided to expand the course participant basis to include nurses wishing to become pain trustees. Thus the course participants include 26 physicians and 12 nurses. We have extended the Pain Trustee Course to 120 hours due to the need to add additional material.

The second-year diploma course will run again next academic year, starting October 2014.
